# 3′-Sialyllactose prebiotics prevents skin inflammation via regulatory T cell differentiation in atopic dermatitis mouse models

**DOI:** 10.1038/s41598-020-62527-5

**Published:** 2020-03-27

**Authors:** Li-Jung Kang, Eunjeong Oh, Chanmi Cho, HoKeun Kwon, Choong-Gu Lee, Jimin Jeon, Hyemi Lee, Sangil Choi, Seong Jae Han, Jiho Nam, Chi-une Song, Hyunho Jung, Hye Young Kim, Eun-Jung Park, Eun-Ju Choi, Jooyoung Kim, Seong-il Eyun, Siyoung Yang

**Affiliations:** 1 Department of Biomedical Sciences, Ajou University Graduate School of Medicine, Suwon, 16499 Republic of Korea; 2 Department of Pharmacology, Ajou University School of Medicine, Suwon, 16499 Republic of Korea; 3 CIRNO, Sungkyunkwan University, Suwon, 16419 Republic of Korea; 4 Department of Microbiology and Immunology, Yonsei University College of Medicine, Seoul, 03722 Korea; 5 Korea Institute of Science & Technology (KIST) Gangneung Institute of Natural Products, Gangwon-do, 25451 Republic of Korea; 6 Department of Life Science, Chung-Ang University, Seoul, 06974 Republic of Korea; 7 Synovizen Inc, Seoul, 06621 Republic of Korea; 8 Laboratory of mucosal immunology, Department of Biomedical Science, Seoul National University College of Medicine, Seoul, 03080 Republic of Korea; 9 East-West Medical Research Institute, Medical Science Research Institute, Kyung Hee University, Seoul, 02447 Republic of Korea; 10 Department of Physical Education, College of Education, Daegu Catholic University, Gyeongsan, 38430 Republic of Korea; 11 Department of Anatomy, School of Medicine, Kyungpook National University, Daegu, Republic of Korea

**Keywords:** Autoimmunity, Immunosuppression

## Abstract

3′-Sialyllactose (3′-SL), a natural prebiotic, maintains immune homeostasis and exerts anti-inflammatory and anti-arthritic effects. Although regulatory T cells (Tregs) prevent excessive inflammation and maintain immune tolerance, the effect of 3′-SL on Treg regulation is unclear. This study aimed to investigate the effect of 3′-SL on Treg responses in atopic dermatitis (AD) pathogenesis. Oral administration of 3′-SL reduced AD-like symptoms such as ear, epidermal, and dermal thickness in repeated topical application of house dust mites (HDM) and 2,4-dinitrochlorobenzene (DNCB). 3′-SL inhibited IgE, IL-1β, IL-6, and TNF-α secretion and markedly downregulated AD-related cytokines including IL-4, IL-5, IL-6, IL-13, IL-17, IFN-γ, TNF-α, and Tslp through regulation of NF-κB in ear tissue. Additionally, *in vitro* assessment of Treg differentiation revealed that 3′-SL directly induced TGF-β-mediated Treg differentiation. Furthermore, 3′-SL administration also ameliorated sensitization and elicitation of AD pathogenesis by suppressing mast cell infiltration and production of IgE and pro-inflammatory cytokines in mouse serum by mediating the Treg response. Furthermore, *Bifidobacterium* population was also increased by 3′-SL administration as prebiotics. Our data collectively show that 3′-SL has therapeutic effects against AD progression by inducing Treg differentiation, downregulating AD-related cytokines, and increasing the *Bifidobacterium* population.

## Introduction

Prebiotics are substances that improve the intestinal environment as a nutrient source for probiotics that help the growth of intestinal microbiota^1^. Prebiotics are often composed of carbohydrates such as oligosaccharides, most of which are in the form of dietary fibre^2^. Probiotics have a beneficial effect on the intestinal environment, prevent the growth of harmful bacteria in the intestine, improve immunity, ameliorate skin diseases such as atopic dermatitis (AD) and psoriasis, and inhibit metabolic syndrome^3^. There are hundreds of beneficial bacteria that live in the intestine, but very few strains can be cultured outside the gut. Therefore, the only way to increase the number of desired strains without external supplementation is to use prebiotics. Notably, these beneficial microbiotas are known to be closely related to many autoimmune diseases such as AD.

AD is a chronic inflammatory skin disease common among infants and children and is characterized by itching erythema and thick skin caused by immune disruption, genetic defects, and environmental factors^4^. In AD, Th2 cell-mediated immune responses are more predominant than Th1-mediated immune responses and they play an important role in the pathogenesis^5^. When foreign antigens penetrate the damaged skin barrier, dendritic cells recognize the antigen and activate Th2 cells. In activated Th2 cells, IL-4, IL-5, IL-13, and atopic-related cytokines including IL-17, and Tslp are secreted, and B cells are activated by IL-4 to secrete IgE^5–7^.

Mast cells are the key effector cells causing allergic reactions and are typically activated by IgE receptors, and undergo degranulation, wherein various inflammatory substances are released, e.g., histamine, serotonin, and tumor necrosis factor-alpha (TNF-α)^8,9^. Furthermore, mast cells synthesize and release various cytokines, including IL-4. These cytokines promote inflammation and facilitate intradermal penetration of more inflammatory cells^8,10^.

Regulatory T cells (Tregs) regulate allergic reactions and significantly contribute to immunosuppression and immune tolerance^11^. Tregs markedly express the forkhead box p3 transcription factor (Foxp3), encoded by X-chromosomal gene *FOXP3*^12^. A dysfunctional Treg leads to autoimmune diseases, including atopic dermatitis, systemic lupus, and asthma^12–14^. Numerous studies have shown that the depletion of Tregs results in an autoimmune phenotype, increasing the production of Th2-related cytokines, and elevating serum IgE levels in mice^13–15^. Both an imbalance in Th1 and Th2 cells and aberrant immune regulation caused by Tregs constitute an important mechanism underlying the pathogenesis of AD^16^. Indeed, it has been suggested that Tregs reside in the skin, contributing to immune surveillance^17,18^. Rapamycin and metformin regulate Tregs through Treg expansion in tissues; hence, these compounds are used as therapeutic agents for autoimmune diseases^19,20^. However, because some of the side effects of these drugs include lactic acidosis, accelerated cataract formation, gastrointestinal intolerance, and erythema, new substances are required^21,22^. There has been growing interest in therapy for autoimmune diseases, including AD, based on probiotics and prebiotics through the induction of Treg differentiation. Prebiotics increase immunological tolerance via expansion of the intestinal microbiota, which induces the differentiation of Tregs and promotes suppressive activities through cell surface receptors such as GPCR (GPR45 and GPR109A).

The prebiotic 3′-SL is abundant in human milk and consists of N-acetylneuraminic acid linked to the galactosyl subunit of lactose^23^. Numerous studies have reported on the beneficial effects of 3′-SL for inflammation and immune homeostasis via changing the intestinal microbiota profiling^24,25^. Moreover, 3′-SL ameliorates the progression of rheumatoid arthritis by downregulating chemokines and cytokines and alleviates osteoarthritis by stimulating cartilage regeneration and protecting cartilage from destruction^26,27^. However, the effect of 3′-SL on AD pathogenesis and its regulation of Treg responses are largely unknown. Accordingly, this study aimed to investigate the effect of 3′-SL on Treg responses in AD pathogenesis.

## Results

### 3′-SL alleviates experimentally-induced AD progression by preventing sensitization and elicitation

**Figure 1:**
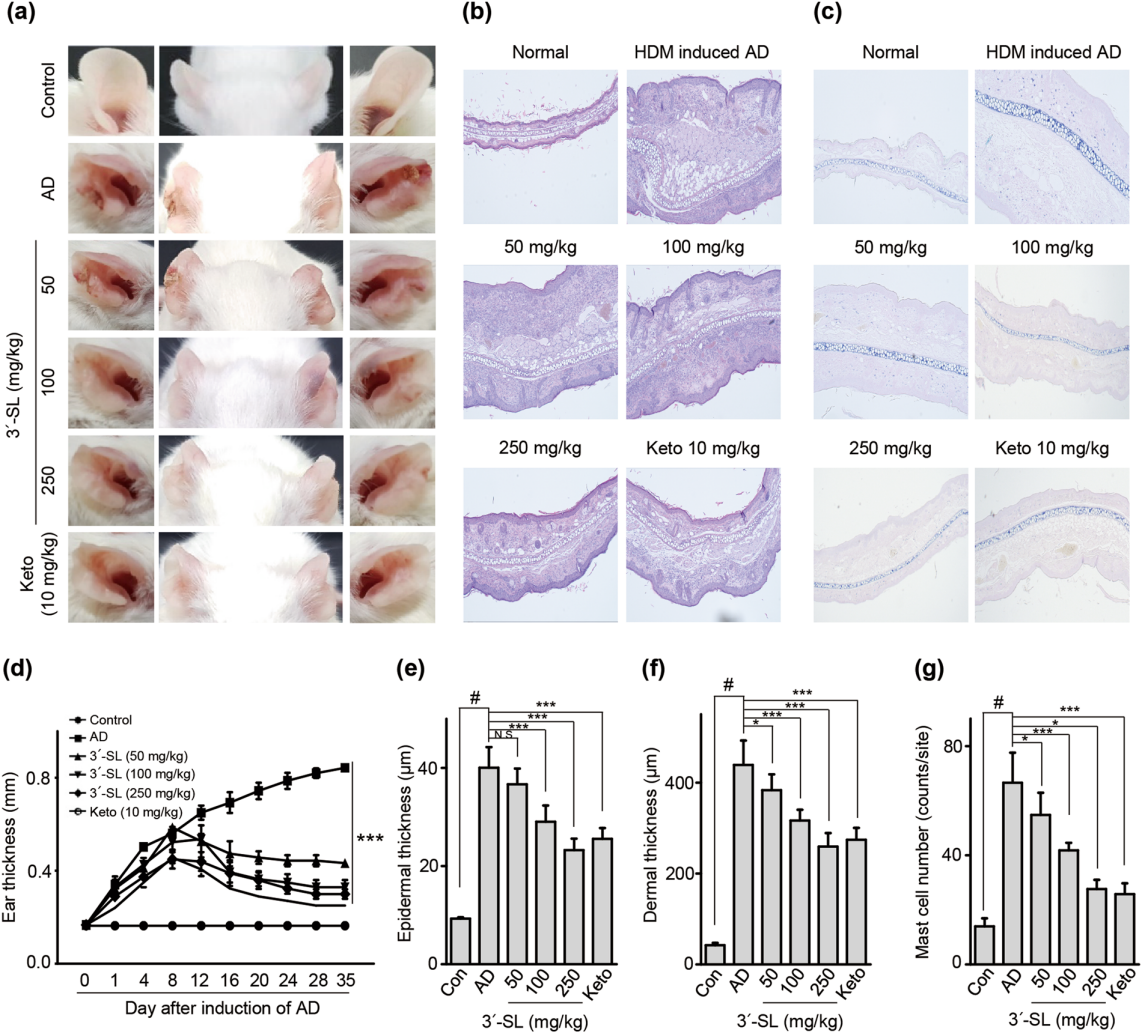
Therapeutic effect of 3′-sialyllactose (3′-SL) after elicitation in HDM-induced atopic dermatitis (AD) mouse model. Oral administration of 3′-SL and or Ketotifen as positive control done at 2-day intervals for 4 weeks after elicitation stage. (**a**) Variation in ear thickness during the course of HDM-induced AD. Atopic episodes during the experiment are shown as photographs. (**d**) Variation in ear thickness from 35 d before the experiment to the end of the experiment. Microphotographs of sections of the left ear 35 d after the onset of AD. The sections were stained with haematoxylin and eosin (H&E) (**b**) and Toluidine Blue (**c**). Original magnification was 100×. (**e)** Epidermal and (**f**) dermal thickness was quantified from H&E-stained microphotographs. (**g**) The number of infiltrating mast cells in the ear sections, as determined through Toluidine Blue staining. At least three randomly selected sites were analysed for each cell count experiment. Data are presented as mean ± SD values for each group (n = 6). ^#^*P* < 0.05 between the HDM treated groups and the control group; *P < 0.05, **P < 0.01, ***P < 0.001 in comparison to HDM^-^treated group.

**Figure 2:**
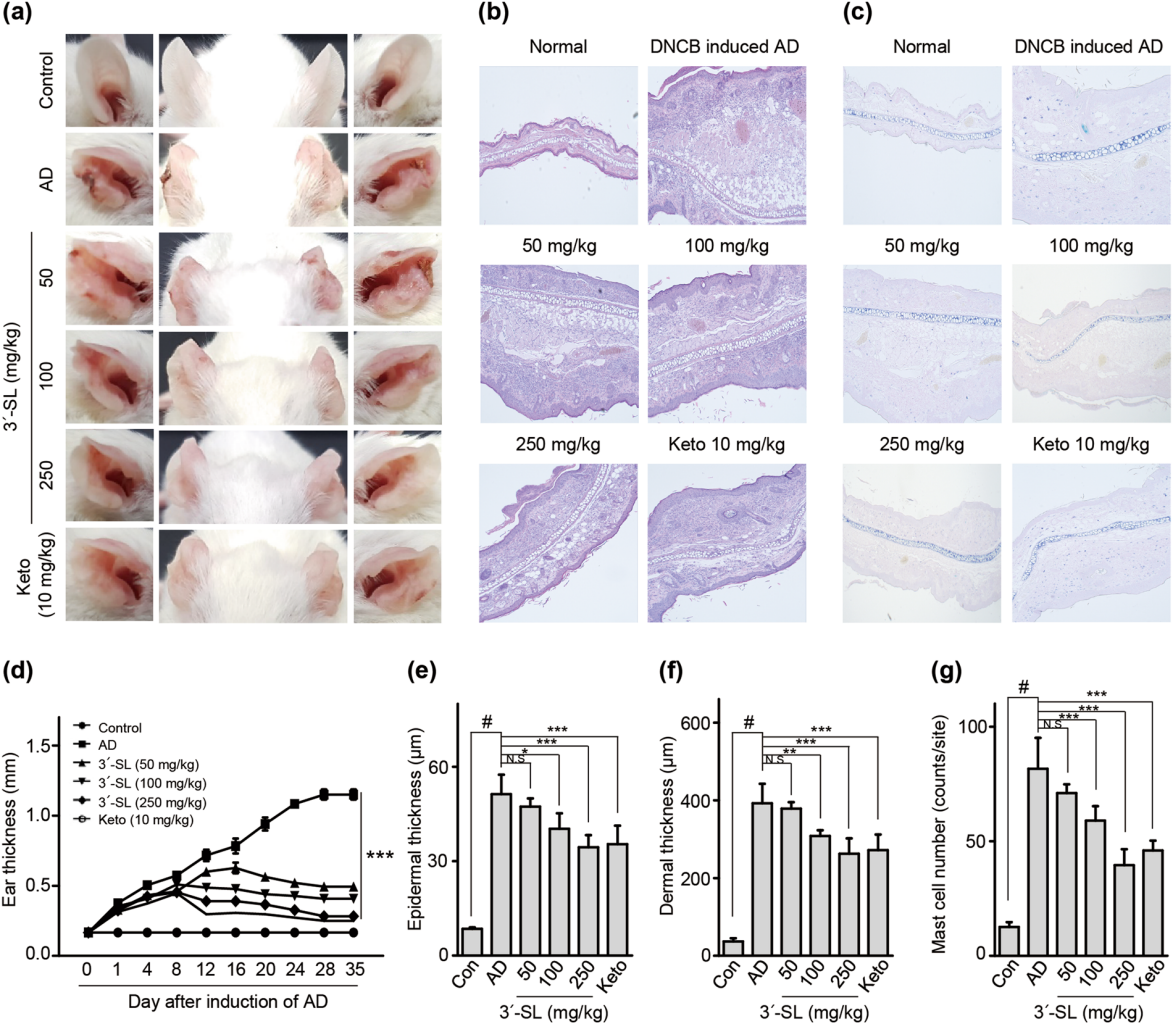
Therapeutic effect of 3′-sialyllactose (3′-SL) after elicitation in 1% DNCB-induced atopic dermatitis (AD) mouse model. Oral administration of 3′-SL and or Ketotifen was done at 2-day intervals for 4 weeks after elicitation stage. (**a**) Variation in ear thickness during the course of 1%DNCB-induced AD. Atopic episodes during the experiment are shown as photographs. (**d**) Variation in ear thickness from 35 d before the experiment to the end of the experiment. Microphotographs of sections of the left ear 35 d after 1% DNCB induced AD. The sections were stained with haematoxylin and eosin (H&E) (**b**) and Toluidine Blue (**c**). Original magnification was 100×. (**e**) Epidermal and (**f**) dermal thickness was quantified from H&E-stained microphotographs. (**g**) The number of infiltrating mast cells in the ear sections, as determined through Toluidine Blue staining. At least three randomly selected sites were analysed for each cell count experiment. Data are presented as mean ± SD values for each group (n = 6). ^#^*P* < 0.05 between the 1% DNCB -treated groups and the control group; *P < 0.05, **P < 0.01, ***P < 0.001 in comparison to 1% DNCB -treated group.

Major triggering allergens are those of the *Dermatophagoides farinae* and *D*. *pteronyssinus* house dust mites (HDM), and 95 percent of human patients with AD display serum levels of HDM-specific IgE^28^. To determine the effect of 3′-SL on AD progression, experimental AD was induced via treatment of mouse ears with HDM at 2-day intervals (Fig. 1, Supplementary Fig. 1b,c), or by treatment with 1% 2,4,-dinitrochlorobenzene (DNCB) at 7-day intervals (Fig. 2, Supplementary Fig. 1d,e). Furthermore, we checked whether 3′-SL prevents allergic sensitization, elicitation, or both. Firstly, 3′-SL was orally administered after elicitation phase (Fig. 1, Supplementary Fig. 1b) or sensitization phase (Supplementary Fig. 1d, 2) in HDM-induced AD mice model. Although mouse-ear thickness increased upon treatment with HDM, 3′-SL significantly decreased the ear thickness in both elicitation phase (Figs. 1a, 2d) and sensitization (Supplementary Fig. 2a,d). Furthermore, after elicitation (Fig. 1) or sensitization stage, macroscopic analysis revealed that HDM induced AD lesions, including erythema, oedema, scaling, and bleeding, which were suppressed upon administration of 3′-SL (Fig. 1a, Supplementary Fig. 2d). Histological changes were analysed by haematoxylin-eosin, and Toluidine Blue staining, which indicated that epidermal and dermal thickness increased upon HDM treatment, and intradermal mast cell infiltration significantly increased in comparison with the control. However, these HMD-mediated changes were markedly decreased by 3′-SL oral administration (Fig. 1c,e–g Supplementary Fig. 2c,e–g).

1% DNCB induced AD also causes allergic contact dermatitis with both sensitization and elicitation phases. As shown in Fig. 2 and Supplementary Fig. 3, 1% DNCB-induced AD phenotypes were also decreased by 3′-SL in sensitization and elicitation phases. Together, these results suggested that 3′-SL suppresses HDM and 1% DNCB-induced AD lesions by decreasing epidermal and dermal thickness and mast cell infiltration in both sensitization and elicitation phase.

### 3′-SL inhibits the production of IgE and pro-inflammatory cytokines in AD-induced mouse serum

**Figure 3:**
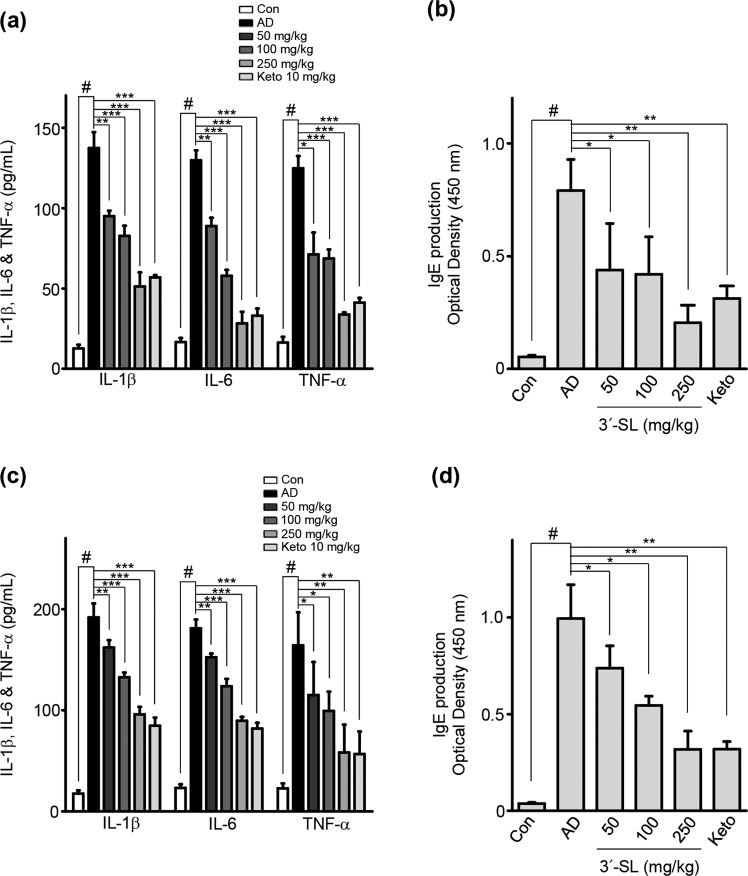
The systemic effect of 3′-sialyllactose (3′-SL) on the secretion of IL-1β, IL-6, TNF-α, and IgE in atopic dermatitis (AD) induced mice serum. Secretion of IL-1β, IL-6, and TNF-α and IgE level in the sera of HDM (**a**,**b**) and 1% DNCB (**c**,**d**) induced mice after elicitation with administered PBS, 3′-SL, and ketotifen as positive control. Serum samples were harvested on day 35 from mice with AD and those administered with 3′-SL, and ketotifen. Secreted IgE and cytokine levels were measured via ELISA. Data are presented as mean ± SD values of each group (n = 6). ^#^*P* < 0.05 between the HDM (**a**,**b**) or 1% DNCB (**c**,**b**)-treated groups and the control group; *P < 0.05, **P < 0.01, ***P < 0.001 in comparison to 1% DNCB or HDM-treated group.

Human AD patients shown high level of IgE and expressed various pro inflammatory cytokines^28^. Therefore, To determine the mechanism underlying 3′-SL-mediated inhibition of AD progression, we used ELISA to measure plasma levels of IgE and pro-inflammatory cytokines in HDM and 1% DNCB induced AD mice with 3′-SL oral administration. Serum IgE levels in the mice decreased upon 3′-SL administration (Fig. 3b,d) Furthermore, Plasma IL-1β, IL-6, and TNF-α levels were decreased in 3′-SL-treated mice with AD (Fig. 3a,c). These results suggest that 3′-SL suppresses AD by reducing the production of IgE level and pro-inflammatory cytokines.

### 3′-SL blocks HDM and 1% DNCB-induced AD pathogenic cytokines via NF-κB inactivation in the ear

**Figure 4:**
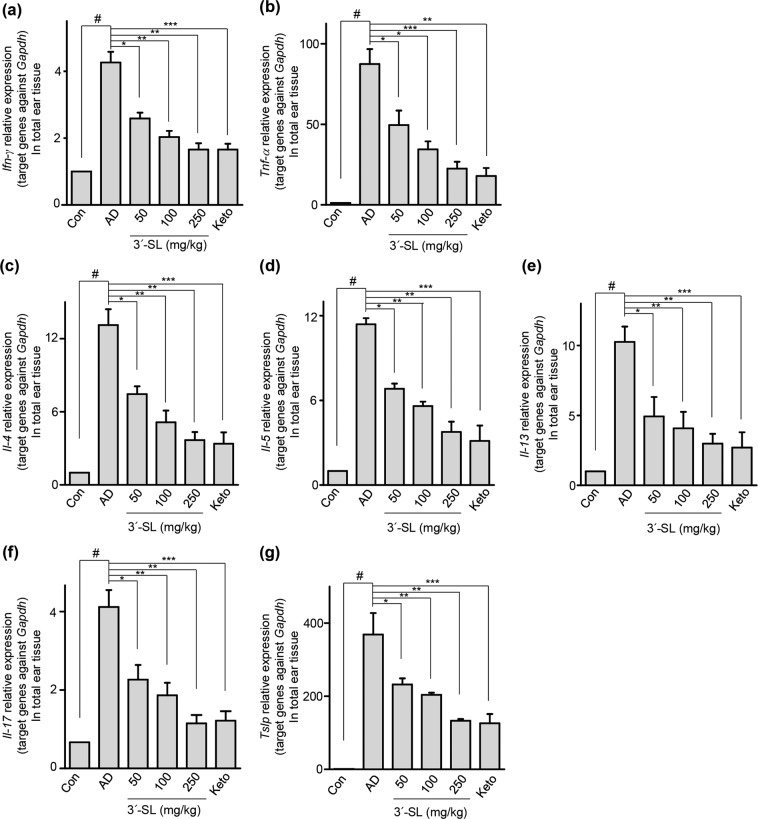
3′-Sialyllactose (3′-SL) protected house dust mite (HDM)-induced atopic dermatitis (AD) progress after onset of elicitation by downregulating pro-inflammatory cytokines in the AD-induced ear tissue of mice. Effect of 3′-SL on the expression of Th1 cytokines (**a**) IFN-gamma and (**b**) TNF-α; and Th2 cytokines (**c**) IL-4 (**d**) IL-5 and (**e**) IL-13; and other AD-related cytokines (**f**) IL-17 and (**g**) Tslp in HMD-inducecd ear tissue. The ears were excised on day 35, total RNA was isolated, and quantitative reverse transcriptase PCR analysis was performed. Data are presented as mean ± SD values for each group (n = 6). ^#^*P* < 0.05 between the HDM-treated and control group; *P < 0.05, **P < 0.01, ***P < 0.001 in comparison to HDM^-^treated group.

To investigate the mechanism underlying 3′-SL-mediated suppression of HDM and 1% DNCB-induced AD, we determined the transcript levels of AD-related inflammatory cytokines in the treated mouse ear tissue by qRT-PCR analysis (Fig. 4 and Supplementary Fig. 4). Th1 type cytokines (IFN-γ, TNF-α) and Th2 type cytokines (IL-4, IL-5, IL-13) were upregulated in HDM- and 1% DNCB-induced mice ear tissue. However, oral administration of 3′-SL (100 and 250 mg/kg) inhibited expression of Th1 cytokines (Fig. 4a and Supplementary Fig. 4a) IFN-gamma and (Fig. 4b and Supplementary Fig. 4b) TNF-α; and Th2 cytokines (Fig. 4c and Supplementary Fig. 4c) IL-4 (Fig. 4d and Supplementary Fig. 4d) IL-5 and (Fig. 4e and Supplementary Fig. 4e) IL-13; and other AD-related cytokines (Fig. 4f and Supplementary Fig. 4f) IL-17 and (Fig. 4g and Supplementary Fig. 4g) Tslp, were also downregulated by oral administration of 3′-SL in the AD induced mice. These results indicate that 3′-SL downregulates the production of cytokines produced by a T-cell subset in the ear tissue of AD mice.

NF-κB is a transcriptional regulator involved in various immune responses, including skin inflammatory diseases^29^. Many studies have reported that AD-related pro-inflammatory cytokines are regulated by NF-κB^29,30^. Therefore, we investigated whether 3′-SL blocks the intranuclear translocation of p65 via immunofluorescence staining. As shown in Supplementary Fig. 5, nuclear localization of p65 in the ear tissue of AD mice was suppressed via oral administration of 3′-SL in a dose dependent manner. These results indicate that 3′-SL suppressed AD-related pro-inflammatory cytokines by blocking intranuclear p65 translocation and inhibiting NF-κB activation.

### 3′-SL alleviates the systemic immune response by inhibiting T cell activation

**Figure 5:**
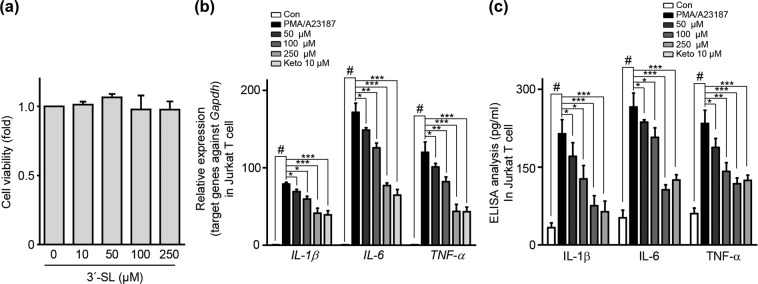
The role of 3′-sialyllactose (3′-SL) in T cell activation. (**a**) The effect of 3′-SL on Jurkat T cell viability was determined by WST-1 assay after culturing Jurkat T cells with various concentrations of 3′-SL. (**b**) Jurkat T cells were treated with PMA/A23187, 3′-SL and Ketotifen as immune modulator for 24 h at the indicated time points. Pro-inflammatory cytokine (TNF-α, IL-6, and IL-1β) mRNA expression levels were determined via qRT-PCR analysis. (**c**) Secretion of pro-inflammatory cytokines was assessed using ELISA. ^#^*P* < 0.05 between the PMA/A23187 treated and control group; *P < 0.05, **P < 0.01, ***P < 0.001 between the PMA/A23187-treated group and 3′-SL treated groups.

Various studies have reported that AD progresses to systemic diseases, including autoimmune disorder, inflammatory bowel disease, and metabolic disease^31,32^. Therefore, we investigated the expression of pro-inflammatory cytokine expression and production in Jurkat T cells. We initially investigated whether 3′-SL is cytotoxic to Jurkat T cells. As shown in Fig. 5a, treatment of Jurkat T cells with 3′-SL at various concentrations for 24 h did not result in cytotoxic effects. Thus, an *in vitro* analysis was performed wherein Jurkat T cells were treated with 0 to 250 μM 3′-SL, in the presence of stimulant PMA/A23187 for 24 h, and then the transcript levels and production of pro-inflammatory cytokines IL-1β, IL-6, and TNF-α were assessed via qRT-PCR and ELISA. As shown in Fig. 5b,c, upregulation and production of IL-1β, IL-6, and TNF-α by PMA/A23187 were drastically decreased by 3′-SL in a dose-dependent manner. These results suggest that 3′-SL suppresses pro-inflammatory cytokines by inhibiting T cell activation.

### 3′-SL induced regulatory T cell differentiation

**Figure 6:**
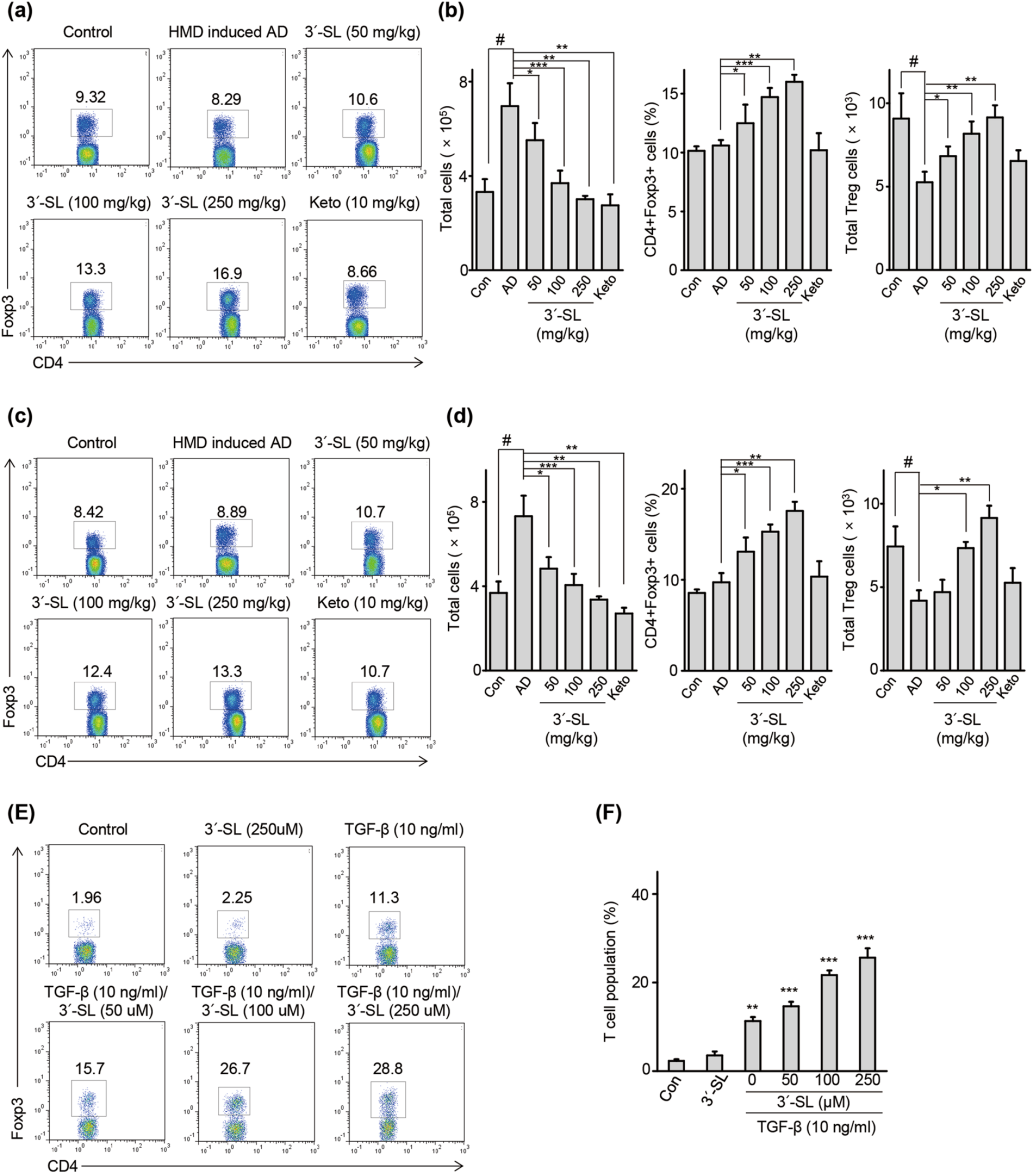
3′-Sialyllactose (3′-SL) regulates Treg differentiation. Draining lymph cells isolated from house dust mite (HMD) treated mice followed by treatment with or without 3′-SL after elicitation stage (**a**,**b**) and sensitization stage (**c**,**d**) were analysed via flow cytometry using CD4 and Foxp3-specific antibodies. The Treg population in total draining lymph node cells were measured. The total cell number (**b**,**d**; left panel), frequency of Treg cells (**b**,**d**; middle panel), and total Treg number (**b**,**d**; right panel) were determined. (**e**,**f**) CD4 + T cells were isolated and cultured for 3 days with TGF-β and 3′-SL on surfaces pre-coated with anti-CD3/anti-CD28 antibody. Cultured T cells were harvested and stained with anti-CD4 and anti-Foxp3 antibodies. Percentages of Foxp3-positive populations were analysed via FACS. Data are presented as mean ± SD values for each group (n = 6). ^#^*P* < 0.05 between HDM-treated groups and the control group; *P < 0.05, **P < 0.01, ***P < 0.001 between the HDM-treated group and 3′-SL or Ketotifen as positive control.

Regulatory T cells suppress excessive inflammatory responses and induce immunologic tolerance to self-antigens, thus inhibiting mast cell activation and IgE production^33^. Consistent with the results mentioned above, 3′-SL suppressed mast cell infiltration and IgE production, and reduced T cell activation through blockade of pro-inflammatory cytokine secretion in Jurkat T cells. Therefore, we next determined whether 3′-SL affects the frequency of Treg cells for both elicitation phase (Fig. 6a,b) and sensitization phase (Fig. 6c,d). Total cell number in the draining lymph nodes increased (Fig. 6b,d; left panel) and the total number of CD4+ FoxP3+ cells decreased (Fig. 6b,d; right panel) in the HDM and 1% DNCB (Supplementary Fig. 6)-induced AD group in comparison with the control group. However, the frequency (Fig. 6a,b; middle panel, Supplementary Fig. 6a,b; middle panel) and total number of CD4 + Foxp3+ cells (Fig. 6b, Supplementary Fig. 6b; right panel) in the draining lymph node was influenced by oral 3′-SL administration. These results suggest that 3′-SL induced the Treg population in the draining lymph node. To verify the effect of 3′-SL on Treg differentiation, we investigated whether 3′-SL treatment enhances Treg differentiation *in vitro*. CD4 + T cells were isolated from the spleen of C57BL/6 mice and cultured in the presence and absence of TGF-β or co-treated with 3′-SL for 3 days. As shown in Fig. 6e,f, co-treatment with 3′-SL and TGF-β significantly increased the Treg population in comparison with the control group and TGF-β-treated group. These results indicate that 3′-SL attenuates progression of AD by promoting differentiation of Tregs.

### 3′-SL administration increases incidence of *Bifidobacterium*

**Figure 7:**
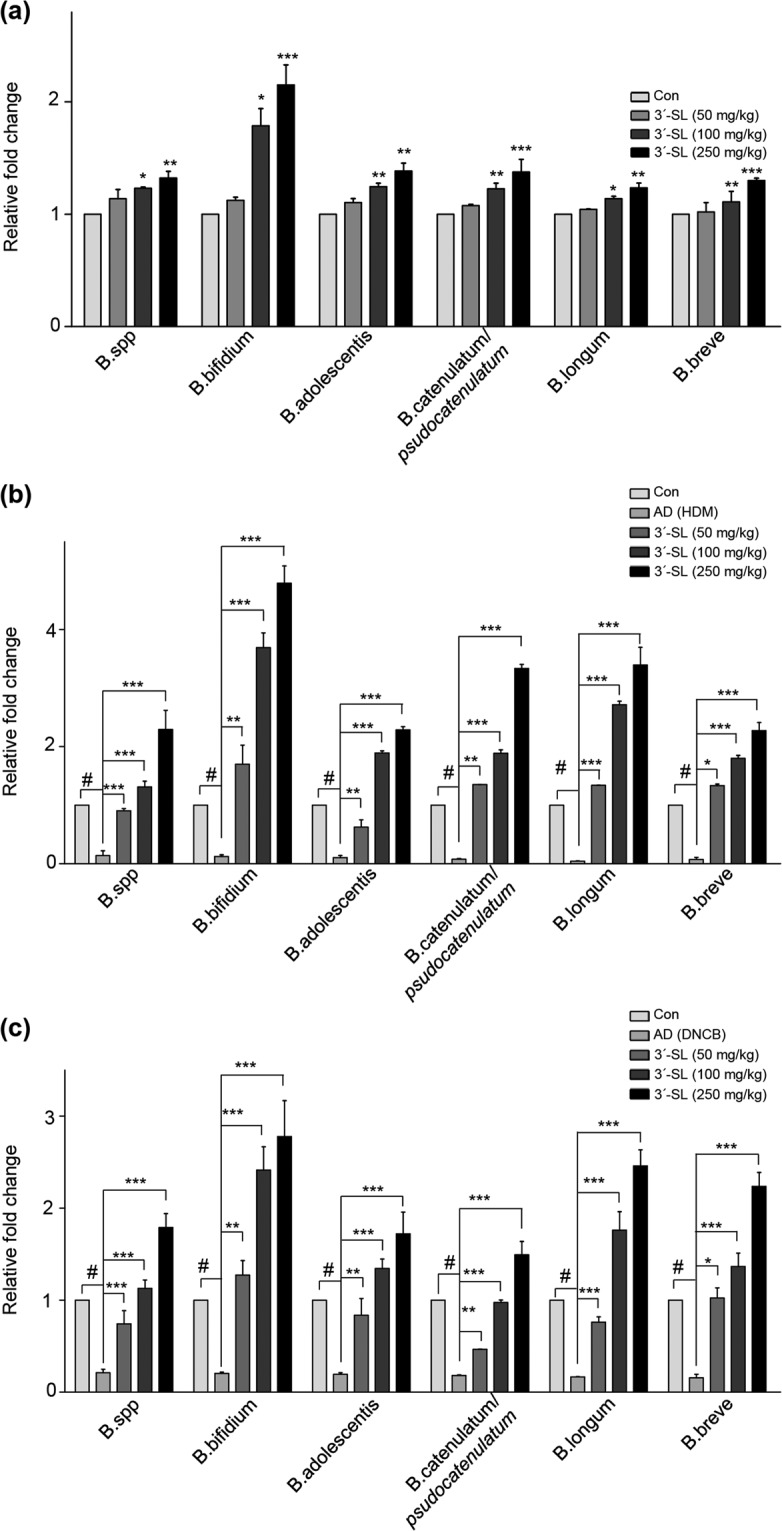
Quantitative analysis of *Bifidobacterium* after oral administration of 3′-sialyllactose (3′-SL). (**a**) The *Bifidobacterium* were determined by qRT-PCR after 3′-SL oral administration of various doses of 3′-SL for 35 day. Quantitated *Bifidobacterium* in house dust mite (HMD) (**b**) and 1% DNCB-induced (**c**) AD progression by qRT-PCR. Mice faeces were collected in control condition and after 35 day of AD progression. Values are shown as relative amount to total bacterial 16 S rDNA measured by the 2^−ΔΔCt^ method. Two independent experiments were performed and data show means ± SEM of 4–6 animals (*n* = 4–6). *P < 0.05.

3′-SL is known to promote the outgrowth of *Bifidobacterium*. Moreover, *Bifidobacterium* is a well-known probiotics for AD^34,35^. To check whether oral administration of 3′-SL modulates *Bifidobacterium* in normal conditions, we first determined levels of *Bifidobacterium* after administration of 3′-SL oral for 35 day in WT mouse. *Bifidobacterium*, especially*, Bifidobacterum bifidium*, were gradually increased by 3′-SL oral administration in dose dependent manner (Fig. 7a). Subsequently, we checked effect of 3′-SL modulate on *Bifidobacterium* in the AD mouse models. Although *Bifidobacterium* were decreased in HDM (Figs. 7b) and 1% DNCB (Fig. 7c) induced AD pathogenesis after elicitation phase, *Bifidobacterium*, especially*, Bifidobacterum bifidium*, were increased by 3′-SL oral administration with rescuing AD phenotypes. These results indicate that 3′-SL can function as a prebiotic to modulate *Bifidobacterium* levels.

## Discussion

Prebiotics are ingredients that have beneficial effects on the host by selectively stimulating intestinal bacteria that can improve host health. Gradually increasing evidence has indicated the beneficial effect of prebiotics on the treatment of patients with AD, which is a chronic inflammatory disease characterized by sensitivity to various allergens and several genetic, environmental, and immunological factors^4^. The common immunological characteristics of patients with AD are mast cell infiltration at the site of the lesion and elevated serum IgE levels^8,36^ Furthermore, Th2 and Th17 immune responses resulting from immune imbalance lead to the secretion of Th2 and Th17 cytokines (e.g., IL-4, IL-5, IL-13, IL17)^5,6,37^. This study shows that 3′-SL attenuates AD pathogenesis by inducing Tregs, thus inhibiting the production of the T cell subset (Th1, Th2, Th17) cytokines (e.g., IL-4, IL-5, IL-13, IL-17, IFN-γ, TNF-α, and Tslp) and reducing T cell activation. Furthermore, 3′-SL reduced the production of IgE in mouse serum and mast cell infiltration in sensitization and elicitation phases to the ear.

Human breast milk contains various bioactive molecules with developmental and protective functions. 3′-SL is one of various human milk oligosaccharides and prebiotics that comprise monosaccharide N-acetylneuraminic acid linked to the galactosyl subunit of lactose at the 3′ position. Particularly, 3′-SL exerts anti-inflammatory effects and supports immune homeostasis^23,24^. Furthermore, 3′-SL promotes the growth and activity of beneficial intestinal bacteria and protects against arthritis. However, no studies have focused on the effect of 3′-SL on skin inflammation.

In AD, T lymphocytes are activated and predominantly infiltrate the site where the AD lesions are located^38^. Th1 cells produce IFN-γ and TNF for macrophage activation, resulting in a delayed immune response. In contrast, Th2 cells produce IL-4, IL-5, and IL-13 to increase IgE synthesis and promote mast cell-induced type 1 hypersensitivity^5,30,37^.

IL-1β is involved in the progression of AD and is a prominent inflammatory cytokine secreted at high concentrations by immune cells and epithelial cells in lesions and the blood of patients with AD. Herein, oral administration of 3′-SL blocked the secretion of IL-1β, IL-6, and TNF-α. Furthermore, the Th1 (IFN-γ, TNF-α) and Th2 (IL-4, IL-5, IL-13) type cytokines in AD lesions in mice were markedly downregulated upon oral 3′-SL administration. Recent evidence suggests that IL-1β regulates the production of Tslp, resulting in AD progression^39^. Tslp is also known as the “master switch” of allergic inflammatory reactions because it affects mast and T cells, leading to skin inflammation, and basophils, and eosinophils leading to both innate and acquired immune responses and the induction of Th2 immune responses^40^. Tslp is an important factor in the pathogenesis of AD^39,41^. In patients with AD, Tslp production is increased in the skin and induces Th2 inflammation^40^. Herein, Tslp was markedly downregulated upon treatment with 3′-SL in mouse ear tissue.

Secretion of Th cell subset cytokines by T cell activation is modulated by transcription factors, including NF-κB, which regulates genes associated with various inflammatory responses^29,30,42^. NF-κB plays an important role in innate/adaptive immune responses and chronic inflammatory responses, particularly in Th1 responses, and regulates inflammation resulting from Th2 cell differentiation and activation^30^. NF-κB is a heterodimeric form of p50/p65, which is present in the cytoplasm and regulates various inflammatory genes upon intranuclear translocation of p65 in various inflammatory conditions^42^. Herein, 3′-SL downregulated proinflammatory cytokines via suppressed T cell activation in Jurkat T cells. In particular, the mechanism underlying the effects of 3′-SL is associated with NF-κB transcriptional regulation via the blockade of intranuclear p65 translocation.

Activated T cells are suppressed by Tregs, which, in turn, are regulated by NF-κB. Tregs have recently been shown to play a role in immune disorders, including AD pathogenesis^18,43^. Tregs secrete immunosuppressive cytokines including IL-10 and TGF-β to suppress excessive inflammatory responses, resulting in blocked secretion of cytokines including IL-1β, IL-6, IL-4, IL-17, TNF-α, and Tslp^13,16,18^. Furthermore, Tregs induce immunological tolerance to self-antigens. Tregs inhibit mast cell and T cell activation during allergic reactions and reduce IgE production by promoting isotype switching of B cells^11,33^. However, high levels of IgE inhibit IL-10 signaling and Treg differentiation in patients with allergy^44^. Many studies have indicated the importance of promoting the differentiation, proliferation, and activity of Treg in allergy patients^11,15^.

3′-SL acts as a prebiotic and leads to changes in the intestinal microbiota profiling. Some studies indicate that oral administration of 3′-SL increases the microbiota, including *Bifidobacterium*^34^. Especially, oral administration of these microbiota attenuated AD progression^35^. Interestingly, oral administration of *Bifidobacterium* induces the Treg population with polysaccharide A or outer membrane vesicles form *Bacteriodes fragilis*^45^. In our study, we checked whether 3′-SL increased *Bifidobacterium* in AD pathogenesis. Although general *Bifidobacterium* were decreased in DNCB and HDM-induced AD pathogenesis, the oral administration of 3′-SL increased most *Bifidobacterium*, especially *Bifidobacterium bifidum*, along with reduced AD pathogenesis.

Herein, the total cell number and frequency in the draining lymph node remained unchanged in the AD group; however, the total number of Tregs was lesser than that in the control group. Moreover, the frequency and total number of Tregs in the draining lymph node was increased upon oral 3′-SL administration. Treg induction reportedly attenuates autoimmune diseases, including AD and T1D^19,46^. Furthermore, *ex vivo* analysis of T cell differentiation revealed that treatment with 3′-SL enhanced the CD4+ Foxp3 + T cell population. In conclusion, this study shows that oral 3′-SL administration reduces the progression of HDM and 1% DNCB-induced AD by reducing T cell activation, enhancing T cell differentiation into Tregs, and increasing the *Bifidobacterium* population. Furthermore, 3′-SL regulates the expression of cytokines produced by Th1 cells (IFN-γ, TNF-α), Th2 cells (IL-4, IL-5, IL-13) and Th17 cells (IL-17) by blocking the intranuclear translocation of p65 in the ear tissue of HDM and 1% DNCB induced AD model mice. Moreover, The doses of 50, 100, and 250 mg/kg in mouse is similar to 17.5, 35.1, and 87.5 mg/kg once every two days in human. If consumed every day, then the sufficient dose would be 8.75, 17.5 and 43.75 mg/kg per day.^54^ The present results suggest that 3′-SL is a potential therapeutic agent for AD and its mechanism involves regulation of Treg differentiation, inflammation, and *Bifidobacterium* population.

## Materials and Methods

### Animals

Eight-week-old male BALB/c and C57BL/6 mice weighing 18–20 g were purchased from DBL, Chungbuk, Korea and housed under standard conditions (temperature, 22–25 °C; 12 h photoperiod). All animal experiments were approved by the Animal Care and Use Committee of the University of Ajou and Global campus, Kyung Hee University (Protocol number: KHGASP-17–040). All *in vivo* experiments were performed according to the guidelines of the National Institutes of Health and in accordance with relevant guidelines and regulations.

### Establishment of an AD mouse model with HDM and 1% DNCB

In the HDM (*Dermatophagoides farinae* extract, Greer Laboratories, Lenoir, NC, USA)-induced atopic dermatitis model, both surfaces of the ear lobes were very gently stripped three times with Tegaderm (Supplementary Fig. 1c). After stripping, 20 μL of HDM (10 mg/mL) was painted on each ear. The application of HDM was repeated 3 times per week for 5 weeks. Experimental AD was induced in 8-week-old male BALB/c mice through serial exposure to *D. farinae* extract (DFE) and DNCB around the ear (Supplementary Fig. 1b), as previously described^47^. 3′-Sialyllactose was purchased from SYNOVIZEN Inc. (Seoul, South Korea). Mice were randomly separated into six groups: control, AD-induction, AD-induction + 50, 100, and 250 mg/kg of 3′-SL and Positive control (Ketotifen 10 mg/kg). Earlobe surfaces were stripped using surgical tape (Nichiban, Tokyo, Japan), and 1% DNCB was applied to each earlobe; DEF (10 mg/mL) was also applied after 4 d on the same site. DEF/DNCB was administered once a week for 5 weeks. 3′-SL (50, 100 and 250 mg/kg) was orally administered for 5 weeks during AD induction. Ear thickness was measured using a dial thickness gauge (Kori Seiki MFG, Co., Tokyo, Japan) after 24 h of DEF/DNCB administration and every 4 days after induction of AD. Blood samples were collected from mice after 35 d and stored at –80 °C until further use.

### Cell cytotoxicity analysis

The cytotoxicity of 3′-SL on Jurkat T cells, cultured in RPMI medium containing 10% foetal bovine serum (FBS), 50 U/mL penicillin, and 50 µg/mL streptomycin, was assessed using the EZ-CyTox Cell viability assay kit (Dogen, Seoul, South Korea) following the manufacturer’s protocol. Briefly, cells were seeded into 96-well culture plate at 1 × 10^4^ cells/well. 3′-SL was supplemented in the cell culture media at 10, 50, 100, and 250 μM, and cells were cultured for 24 h. Thereafter, the WST-1 solution (2-(4-iodophenyl)-3-(4-nitrophenyl)-5-(2,4 disulfophenyl)-2H-tetrazolium) was mixed with serum-free RPMI (1:10 v/v) and added to each well for 2 h in the dark. Cytotoxicity was determined at 450 nm using a microplate reader VICTOR X3 (PerkinElmer, Waltham, MA, USA).

### Quantitative reverse transcriptase PCR (qRT-PCR) analysis

Total RNA was isolated from mouse ear tissue and Jurkat T cells using TRIzol reagent (Molecular Research Center Inc., Cincinnati, OH, USA) in accordance with the manufacturer’s instructions. cDNA was synthesized using 1 µg of total mRNA and amplified with target gene primers (Table S1) via PCR (Intron Biotechnology, Gyeonggi-do, South Korea). Relative mRNA expression levels were determined using the SYBR premix Ex Taq (TaKaRa Bio, Shiga, Japan) and normalized to those of *GAPDH*. To detect *Bifidobacterium*, fresh faecal samples were collected from mice and immediately stored at −150 °C until processing. Faecal DNA was isolated using the FastDNA Spin kit (MP Biomedicals, Santa Ana, CA, USA). PCR amplification was performed with target gene primer (Table S2)^48,49^ via qRT-PCR. Quantification values were calculated by the 2^−ΔΔCt^ method relative to total bacteria 16 S rDNA amplification.

### Enzyme-linked immunosorbent assay (ELISA)

IL-1β, IL-6, TNF-α, and IgE production was quantified using an ELISA kit (Koma Biotech, Seoul, South Korea) in accordance with the manufacturer’s instructions. Absorbance for each cytokine and IgE was determined at 450 nm using a microplate reader (VICTOR X3; PerkinElmer, Waltham, MA, USA).

### Immunofluorescence assays

Mouse-ear tissue was cut into 5-µm-thick sections, which were incubated with rabbit anti-p65 antibody (1:200) (8242; Cell Signaling Technology) overnight at 4 °C. After washing with PBS, the sections were incubated with goat anti-rabbit secondary antibody (ab150081; Abcam, Cambridge, UK) conjugated with Alexa Fluor 488 for 2 h at room temperature. Thereafter, slides were washed with PBS and incubated with DAPI solution (4,6-diamidino-2-phenylindole) (DAPI; Invitrogen, San Francisco, CA, USA) for 10 min at room temperature. Mouse ear tissue was imaged using Axioscan (Zeiss) at the Three-dimensional immune system core facility of Ajou University.

### Antibody staining and flow cytometric analyses

Draining lymph node samples were obtained from BALB/c mice. Single cells were isolated via cervical dislocation, washed with PBS containing 10% FBS, and surface stained for CD4 using FITC-conjugated anti-CD4 antibody (300506; BioLegend, San Diego, CA, USA). Subsequently, cells were resuspended in fixation/permeabilization buffer for 12 h and stained for intracellular Foxp3 using anti-Foxp3 APC antibody (17-5773-82; Thermo Fisher Scientific, Waltham, MA, USA) following the manufacturer’s instructions. Each sample was analysed using a FACS MACSQuant VYB flow cytometer (Bergisch Gladbach, Germany).

### T cell proliferation assay

Spleen samples were obtained from C57BL/6 mice. Single cells were isolated via cervical dislocation; CD4^+^ T cells were negatively selected using Anti-Biotin MicroBeads (130-090-485; Miltenyi). CD4^+^ T cells were re-suspended (10^6^ cells/mL) in complete culture media comprising TexMacs medium supplemented with 10% FBS, 4 mM L-glutamine, 50 U/mL penicillin, and 50 μg/mL streptomycin; the cells were then incubated in 24-well plates pre-coated with 5 μg/mL anti-mouse CD3 monoclonal antibody (100201; Biolegend) and 1 μg/mL anti-mouse CD28 antibody (122022; Biolegend) and treated with 3′-SL (50, 100, 250 μM) and TGF-β (10 ng) for 3, 6, and 9 d. The Treg population was assessed via FACS.

### Statistical analysis

Data are presented as mean ± SD values. Differences between the control and treatment groups were evaluated using one-way ANOVA with Dunnett’s post-hoc multiple comparison tests. Statistical significance was determined at *P* < 0.05. Statistical analysis was performed using GraphPad Prism 5 software (GraphPad, San Diego, CA, USA)^49^.

## Supplementary information

Supplymentary information.
