# Supplementary material for: 3′-Sialyllactose prebiotics prevents skin inflammation via regulatory T cell differentiation in atopic dermatitis mouse models
Source: Sci Rep. 2020 Mar 27;10:5603. doi: 10.1038/s41598-020-62527-5 (PMC7101440; doi:10.1038/s41598-020-62527-5)
Supplement: Supplementary file 1 — Supplymentary information. [file 41598_2020_62527_MOESM1_ESM.pdf]

SUPPLEMENTARY FIGURE 1

3′-Sialyllactose prebiotics prevents skin inflammation via regulatory T cell differentiation in atopic dermatitis mouse models

Li-Jung Kang[1,2,3]*, Eunjeong Oh[1,2,3]*, Chanmi Cho[1,2,3], HoKeun Kwon[4], Choong-Gu Lee[5], Jimin Jeon[1,2,3], Hyemi Lee[1,2,3], Sangil Choi[1,2,3], Seong Jae Han[1,2,3], Jiho Nam[1,2,3], Chi-une Song[6], Hyunho Jung[7], Hye Young Kim[3,8], Eun-Jung Park[9], Eun-Ju Choi[10], Jooyoung Kim[11]†, Seong-il Eyun[6]†, Siyoung Yang[1,2,3]†

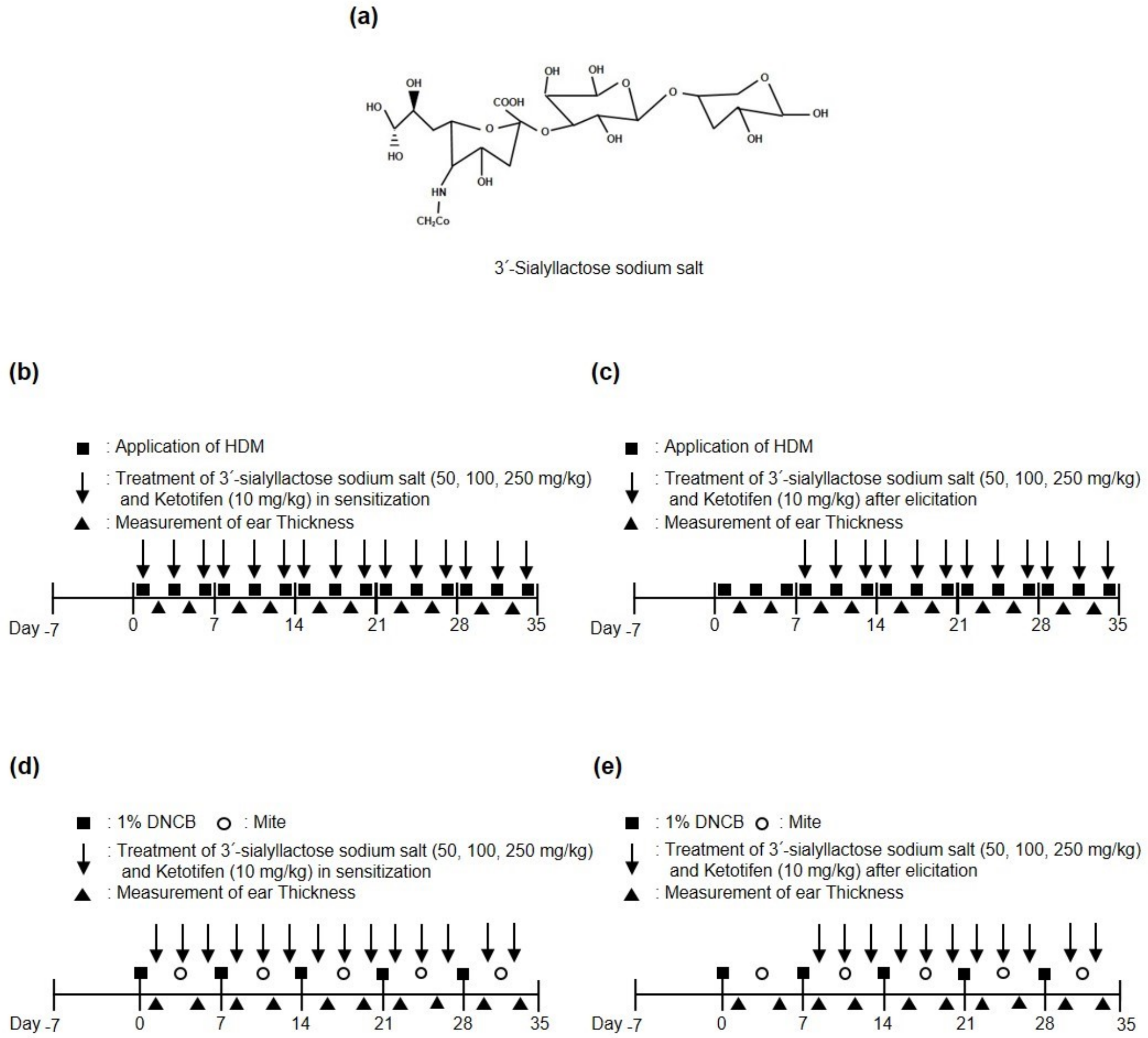

Supplementary Figure. 1

The structure of 3′-sialyllactose (3′-SL) and the protocol for experimental induction of atopic dermatitis (AD). (**a**) Structure of 3′-SL and schematic representation of the protocol for preventing sensitization (**b, d**) and elicitation (**c, e**) in experimental HDM (**b**, **c**) and 1% DNCB (**d**, **e**)-induced AD lesions. Mice were divided into six groups: healthy untreated Controls (Con), mice treated with HDM, or 1% DNCB alone, and mice treated with sodium salt of 3′-SL (50, 100 and 250 mg/kg) or Ketotifen (10 mg/kg) as positive control. Oral administration of 3′-SL and Ketotifen occurred at 2-day intervals for 4 weeks after first treatment of 1% DNCB and HDM (left panel) or elicitation stage (right panel).

SUPPLEMENTARY FIGURE 2

3′-Sialyllactose prebiotics prevents skin inflammation via regulatory T cell differentiation in atopic dermatitis mouse models

Li-Jung Kang[1,2,3]*, Eunjeong Oh[1,2,3]*, Chanmi Cho[1,2,3], HoKeun Kwon[4], Choong-Gu Lee[5], Jimin Jeon[1,2,3], Hyemi Lee[1,2,3], Sangil Choi[1,2,3], Seong Jae Han[1,2,3], Jiho Nam[1,2,3], Chi-une Song[6], Hyunho Jung[7], Hye Young Kim[3,8], Eun-Jung Park[9], Eun-Ju Choi[10], Jooyoung Kim[11]†, Seong-il Eyun[6]†, Siyoung Yang[1,2,3]†

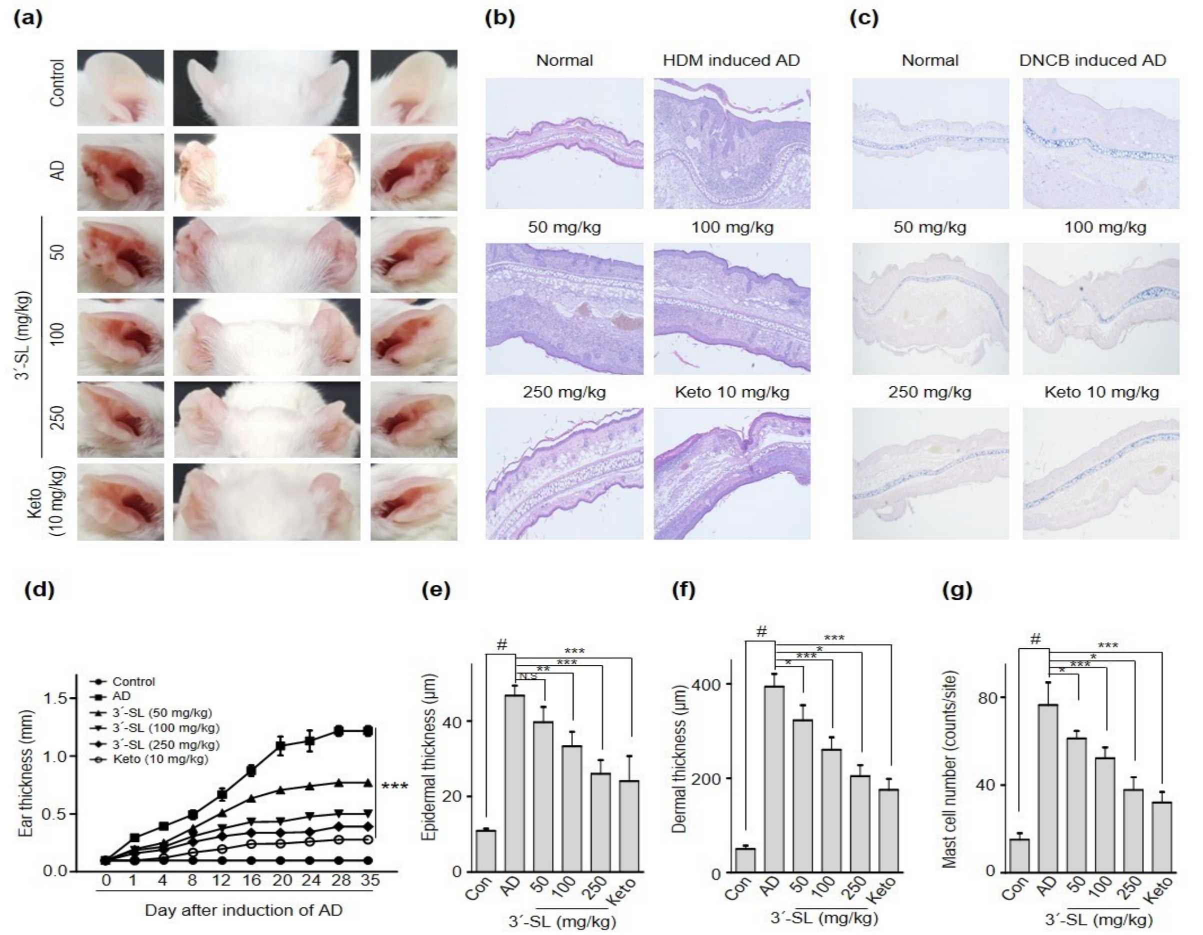

Supplementary Figure. 2

Oral 3′-sialyllactose (3′-SL) administration ameliorated the severity 1% DNCB induced atopic dermatitis (AD). (**a**) Variation in ear thickness during the course of AD. Atopic episodes during the experiment are shown as photographs. (**d**) Variation in ear thickness from 35 d before the experiment to the end of the experiment. Microphotographs of sections of the left ear 35 d after the onset of AD. The sections were stained with haematoxylin and eosin (H&E) (**b**) and Toluidine Blue (**c**). Original magnification was 100×. (**e, f**) Epidermal and dermal thickness was quantified from H&E-stained microphotographs. (**g**) The number of infiltrating mast cells in the ear sections, as determined through Toluidine Blue staining. At least three randomly selected sites were analysed for each cell count experiment. Data are presented as mean ±SD values for each group (n = 6). $^{\#}P < 0.05$ between the 1% DNCB-treated groups and the control group; $^{*}P < 0.05$, $^{**}P < 0.01$, $^{***}P < 0.001$ in comparison to 1% DNCB-treated group.

SUPPLEMENTARY FIGURE 3

3′-Sialyllactose prebiotics prevents skin inflammation via regulatory T cell differentiation in atopic dermatitis mouse models

Li-Jung Kang[1,2,3]*, Eunjeong Oh[1,2,3]*, Chanmi Cho[1,2,3], HoKeun Kwon[4], Choong-Gu Lee[5], Jimin Jeon[1,2,3], Hyemi Lee[1,2,3], Sangil Choi[1,2,3], Seong Jae Han[1,2,3], Jiho Nam[1,2,3], Chi-une Song[6], Hyunho Jung[7], Hye Young Kim[3,8], Eun-Jung Park[9], Eun-Ju Choi[10], Jooyoung Kim[11]†, Seong-il Eyun[6]†, Siyoung Yang[1,2,3]†

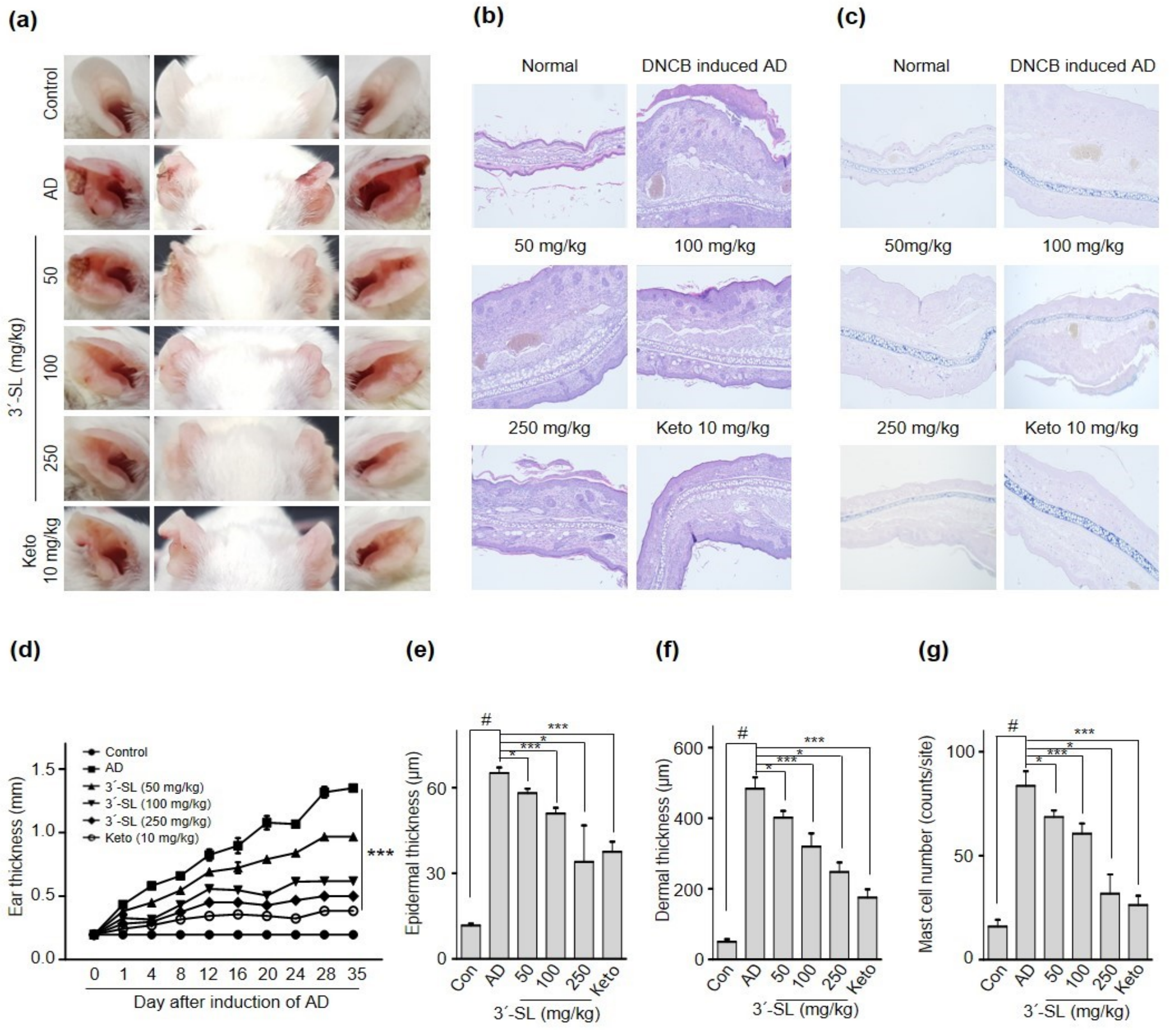

Supplementary Figure. 3

Oral 3′-sialyllactose (3′-SL) administration protected sensitization stage in the 1% DNCB induced atopic dermatitis (AD) mouse model. (**a**) Variation in ear thickness during the course of AD. Atopic episodes during the experiment are shown as photographs. (**d**) Variation in ear thickness from 35 d before the experiment to the end of the experiment. Microphotographs of sections of the left ear 35 d after the onset of AD. The sections were stained with haematoxylin and eosin (H&E) (**b**) and Toluidine Blue (**c**). Original magnification was 100×. (**e, f**) Epidermal and dermal thickness was quantified from H&E-stained microphotographs. (**g**) The number of

infiltrating mast cells in the ear sections, as determined through Toluidine Blue staining. At least three randomly selected sites were analysed for each cell count experiment. Data are presented as mean ± SD values for each group (n = 6). $^{\#}P < 0.05$ between the 1% DNCB treated groups and the control group; $^{*}P < 0.05$, $^{**}P < 0.01$, $^{***}P < 0.001$ in comparison to 1% DNCB-treated group.

SUPPLEMENTARY FIGURE 4

3′-Sialyllactose prebiotics prevents skin inflammation via regulatory T cell differentiation in atopic dermatitis mouse models

Li-Jung Kang[1,2,3]*, Eunjeong Oh[1,2,3]*, Chanmi Cho[1,2,3], HoKeun Kwon[4], Choong-Gu Lee[5], Jimin Jeon[1,2,3], Hyemi Lee[1,2,3], Sangil Choi[1,2,3], Seong Jae Han[1,2,3], Jiho Nam[1,2,3], Chi-une Song[6], Hyunho Jung[7], Hye Young Kim[3,8], Eun-Jung Park[9], Eun-Ju Choi[10], Jooyoung Kim[11]†, Seong-il Eyun[6]†, Siyoung Yang[1,2,3]†

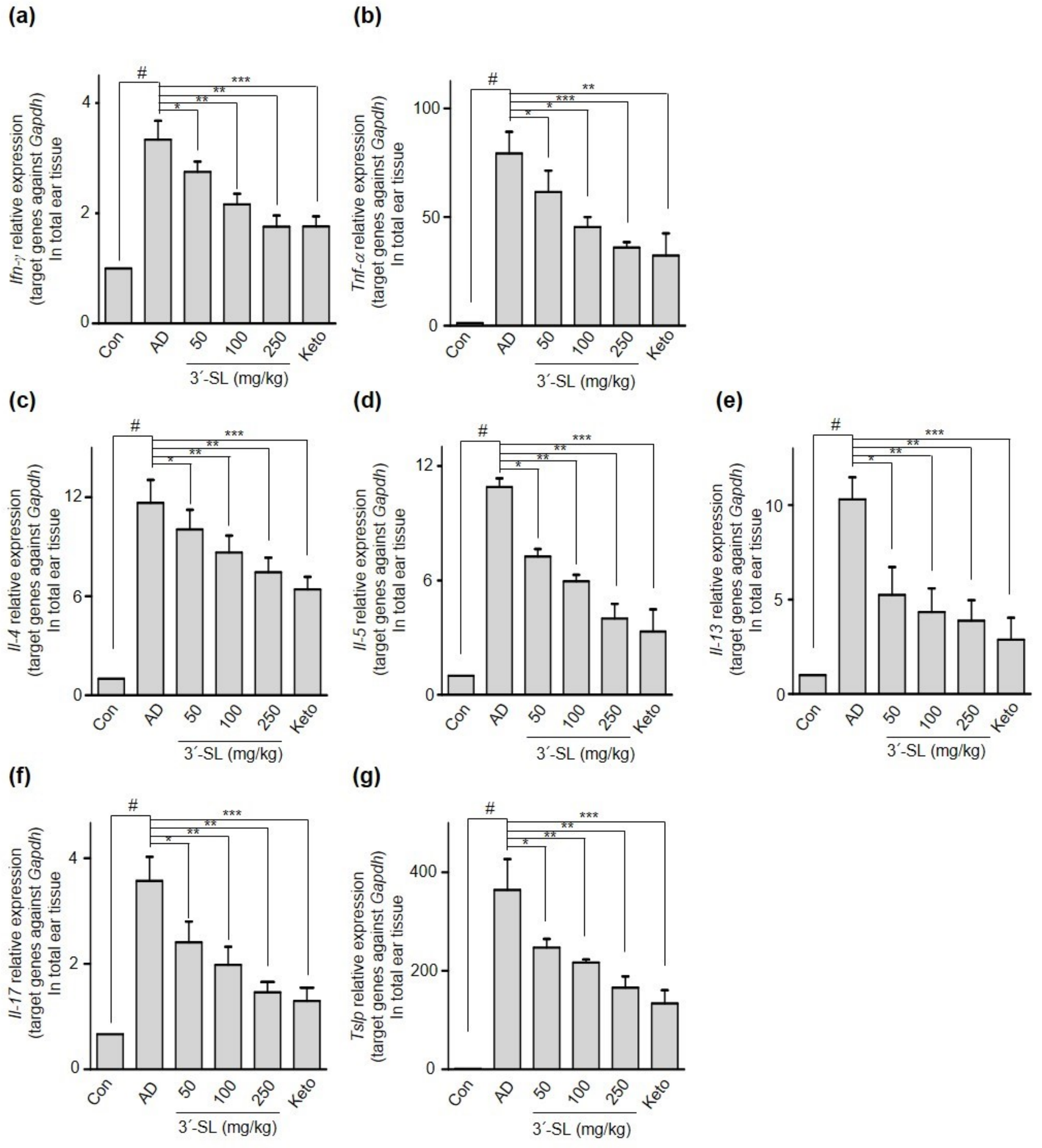

Supplementary Figure. 4

3′-Sialyllactose (3′-SL) protected against 1% DNCB-induced AD progression by downregulation of pro-inflammatory cytokines in the AD induced ear tissue of mice. Effect of 3′-SL on the expression of Th1 cytokines (**a**) IFN-gamma and (**b**) TNF-α; and Th2 cytokines (**c**) IL-4 (**d**) IL-5 and (**e**) IL-13; and other AD-related cytokines (**f**) IL-17 and (**g**) Tslp in 1% DNCB induced mice ear tissue. The ears were excised on day 28, and total RNA was isolated.

The quantitative reverse transcriptase PCR analysis was performed. Data are presented as mean ± SD values for each group (n = 6). Significant differences ($^{\#}P < 0.05$) between the 1% DNCB-treated groups and the control group. Significant differences from the 1% DNCB -treated group were considered as $^{*}P < 0.05$, $^{**}P < 0.01$, $^{***}P < 0.001$.

SUPPLEMENTARY FIGURE 5

3′-Sialyllactose prebiotics prevents skin inflammation via regulatory T cell differentiation in atopic dermatitis mouse models

Li-Jung Kang[1,2,3]*, Eunjeong Oh[1,2,3]*, Chanmi Cho[1,2,3], HoKeun Kwon[4], Choong-Gu Lee[5], Jimin Jeon[1,2,3], Hyemi Lee[1,2,3], Sangil Choi[1,2,3], Seong Jae Han[1,2,3], Jiho Nam[1,2,3], Chi-une Song[6], Hyunho Jung[7], Hye Young Kim[3,8], Eun-Jung Park[9], Eun-Ju Choi[10], Jooyoung Kim[11]†, Seong-il Eyun[6]†, Siyoung Yang[1,2,3]†

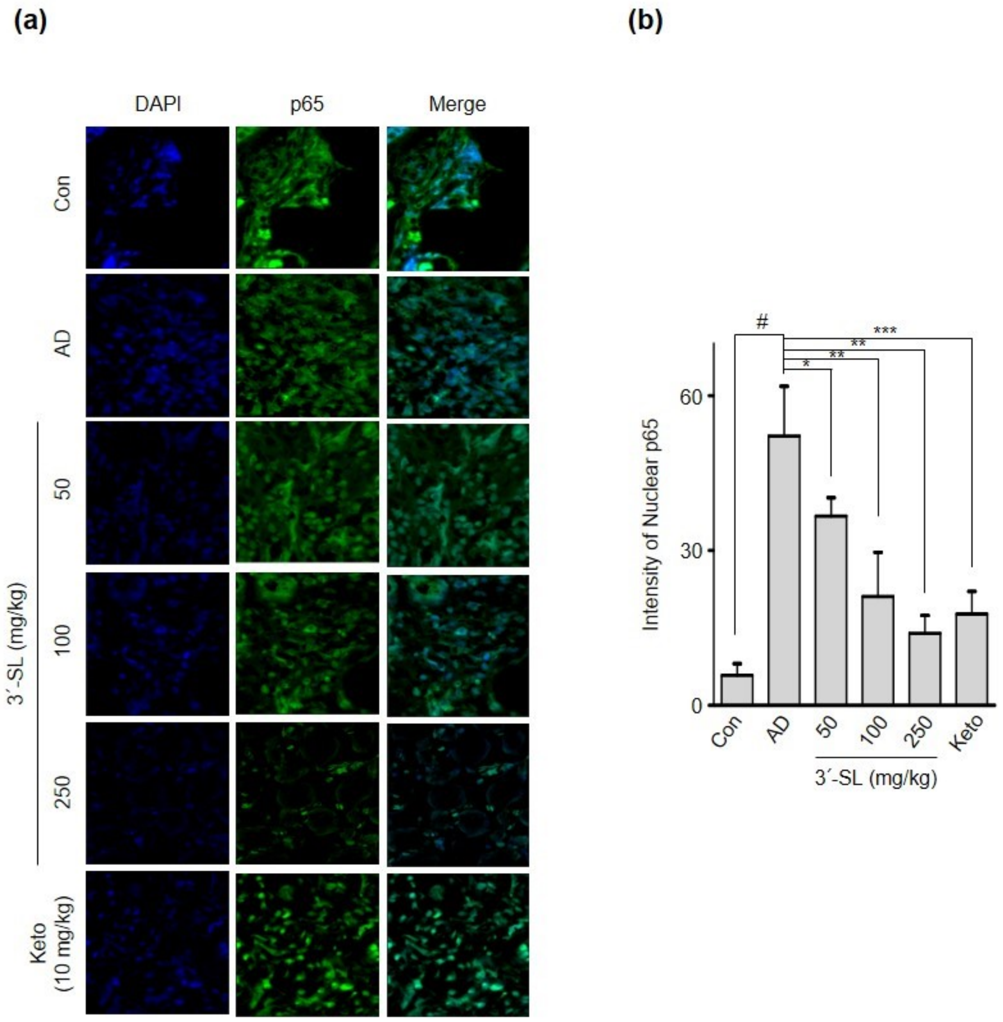

Supplementary Figure. 5

Blockade of NF-κB activity by 3′-sialyllactose (3′-SL) under 1% DNCB treated conditions**.** p65 levels were assessed via immunofluorescence using (**a**) anti-NF-kB p65 (green) antibody in each case, along with DAPI (blue) staining. (**b**) Quantification of p65 expression in mouse ear tissue. Representative images from each treatment set are shown. $^{\#}P < 0.05$ between the 1% DNCB-treated group and the control group; $^{*}P < 0.05$, $^{**}P < 0.01$, $^{***}P < 0.001$ in comparison to 1% DNCB-treated group.

SUPPLEMENTARY FIGURE 6

3′-Sialyllactose prebiotics prevents skin inflammation via regulatory T cell differentiation in atopic dermatitis mouse models

Li-Jung Kang[1,2,3]*, Eunjeong Oh[1,2,3]*, Chanmi Cho[1,2,3], HoKeun Kwon[4], Choong-Gu Lee[5], Jimin Jeon[1,2,3], Hyemi Lee[1,2,3], Sangil Choi[1,2,3], Seong Jae Han[1,2,3], Jiho Nam[1,2,3], Chi-une Song[6], Hyunho Jung[7], Hye Young Kim[3,8], Eun-Jung Park[9], Eun-Ju Choi[10], Jooyoung Kim[11]†, Seong-il Eyun[6]†, Siyoung Yang[1,2,3]†

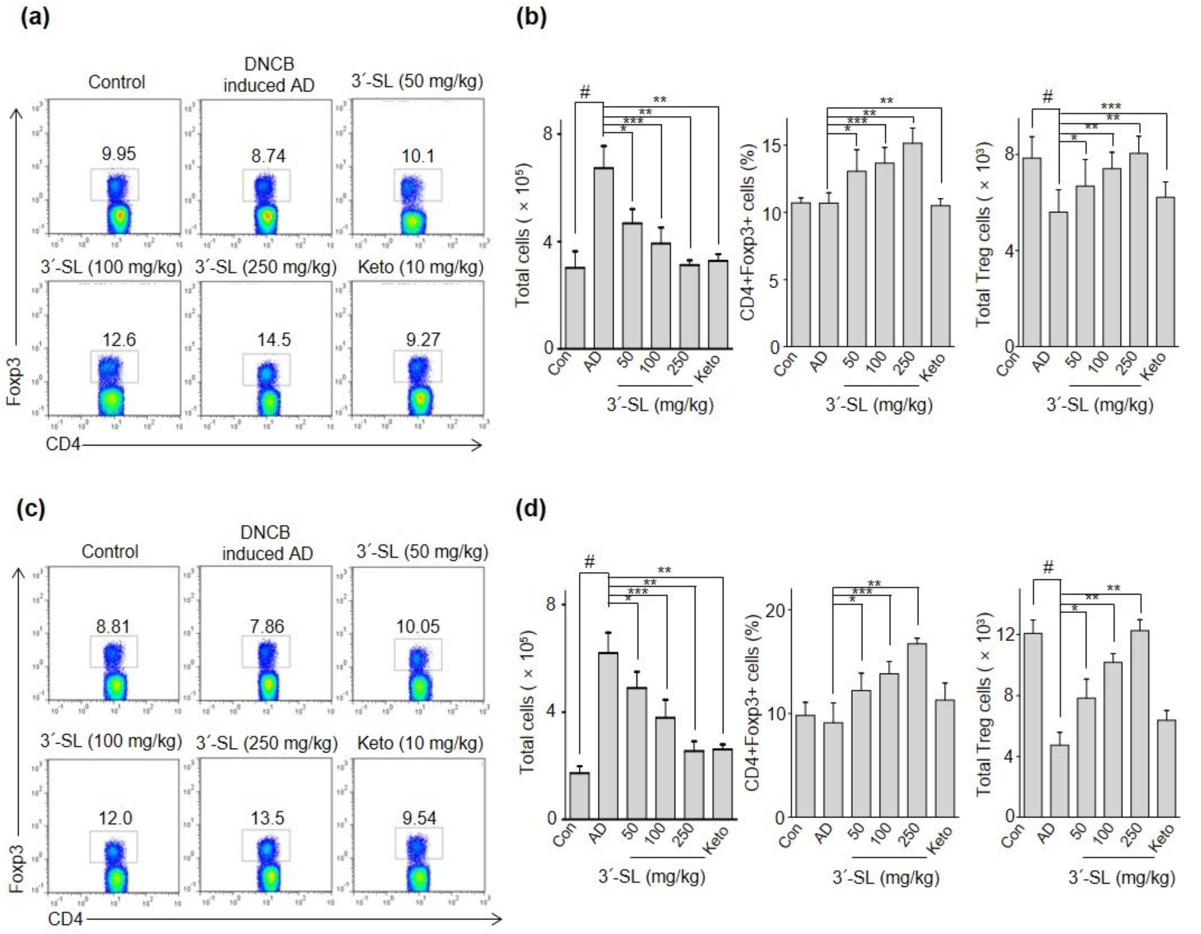

Supplementary Figure. 6

3′-sialyllactose (3′-SL) enhanced Treg differentiation.

Draining lymph cells isolated from 1% DNCB-treated mice with or without after elicitation stage (**a**, **b**) and sensitization stage (**c**, **d**) were analysed via flow cytometry using CD4 and Foxp3-specific antibodies. The Treg population in (**a, c**) total draining lymph node cells were measured. The total cell number (b, d; left panel), percentage of Treg cells (b, d; middle panel), and total Treg number (b, d; right panel) were determined. Data are presented as mean ± SD values for each group (n = 6). $^{\#}P < 0.05$ between the 1% DNCB treated groups and the control group; $^{*}P < 0.05$, $^{**}P < 0.01$, $^{***}P < 0.001$ in comparison to 1% DNCB treated group.

SUPPLEMENTARY TABLE 1

3′-Sialyllactose prebiotics prevents skin inflammation via regulatory T cell differentiation in atopic dermatitis mouse models

Li-Jung Kang[1,2,3]*, Eunjeong Oh[1,2,3]*, Chanmi Cho[1,2,3], HoKeun Kwon[4], Choong-Gu Lee[5], Jimin Jeon[1,2,3], Hyemi Lee[1,2,3], Sangil Choi[1,2,3], Seong Jae Han[1,2,3], Jiho Nam[1,2,3], Chi-une Song[6], Hyunho Jung[7], Hye Young Kim[3,8], Eun-Jung Park[9], Eun-Ju Choi[10], Jooyoung Kim[11]†, Seong-il Eyun[6]†, Siyoung Yang[1,2,3]†

Table. S1. Primer sequences and PCR conditions

Gene	Origin	Strand	Sequence	AT[a] (°C)
Il1b	Mouse	[b]S	5'-TTGACAGTGATGAGAATGACC-3'	65
		[c]As	5'-GCAGGTTATCATCATCATCC-3'	
Ifng	Mouse	S	5'-AAGCCTGTAGCCCACGTCGTA-3'	60
		As	5'-GGCACCACTAGTTGGTTGTCTTTG-3'	
Tnfa	Mouse	S	5'-AAGCCTGTAGCCCACGTCGTA-3'	60
		As	5'-GGCACCACTAGTTGGTTGTCTTTG-3'	
Il4	Mouse	S	5'-ACAGGAGAAGGGACGCCAT-3'	60
		As	5'-GAAGCCGTACAGACGAGCTCA-3'	
Il5	Mouse	S	5'-AGAGTGGGCAATGGAAGGC-3'	60
		As	5'-TTTTGTGGGGTTTTTGCATCTGT-3'	
Il6	Mouse	S	5'-CCGGAGAGGAGACTTCACAG -3'	58
		As	5'- GGAAATTGGGGTAGGAAGGA -3'	
Il13	Mouse	S	5'-GACCATCCCTGGGCATCTCA-3'	60
		As	5'-GCATCTCCCTTCCTCCTCAAC-3'	
Il17	Mouse	S	5'-TCCCCTCTGTCATCTGGGAAG-3'	60
		As	5'-CTCGACCCTGAAAGTGAAGG-3'	
Tslp	Mouse	S	5'-AGGCTACCCTGAAACTGAG-3'	63
		As	5'-GGAGATTGCATGAAGGAATACC-3'	
Gapdh	Mouse	S	5'-TCACTGCCACCCAGAAGAC-3'	55
		As	5'-TGTAGGCCATGAGGTCCAC-3'	

[a]AT, annealing temperature; [b]S, sense; [c]As, antisense

SUPPLEMENTARY TABLE 2

3′-Sialyllactose prebiotics prevents skin inflammation via regulatory T cell differentiation in atopic dermatitis mouse models

Li-Jung Kang[1,2,3]*, Eunjeong Oh[1,2,3]*, Chanmi Cho[1,2,3], HoKeun Kwon[4], Choong-Gu Lee[5], Jimin Jeon[1,2,3], Hyemi Lee[1,2,3], Sangil Choi[1,2,3], Seong Jae Han[1,2,3], Jiho Nam[1,2,3], Chi-une Song[6], Hyunho Jung[7], Hye Young Kim[3,8], Eun-Jung Park[9], Eun-Ju Choi[10], Jooyoung Kim[11]†, Seong-il Eyun[6]†, Siyoung Yang[1,2,3]†

Table. S2. Primer sequences and PCR conditions for bifidobacteriums

Class/Family/ Genus	Species/ Group	Strand	Sequence	AT[a] (°C)
Bifidobacterium	*Spp*	S	5'-GCGTGCTTAACACATGCAAGTC-3'	55
		As	5'-CACCCGTTTCCAGGAGCTATT-3'	
Bifidobacterium	*bifidum*	S	5'- CCACATGATCGCATGTGATTG -3'	55
		As	5'- CCGAAGGCTTGCTCCCAAA -3'	
Bifidobacterium	*adolescentis*	S	5'- CTCCAGTTGGATGCATGTC -3'	55
		As	5'- CGAAGGCTTGCTCCCAGT -3'	
Bifidobacterium	*catenulatum/*	S	5'- CGGATGCTCCGACTCCT -3'	55
	psudocatenulatum	As	5'- CGAAGGCTTGCTCCCGAT -3'	
Bifidobacterium	*longum*	S	5'- GGATGTTCCAGTTGATCGCATGGTC -3'	55
		As	5'-GCATCTCCCTTCCTCCTCAAC-3'	
Bifidobacterium	*breve*	S	5'-TCCCCTCTGTCATCTGGGAAG-3'	55
		As	5'- AGCCGGTGCTTATTCAACGGGTAA -3'	
16s rDNA		S	5'- AATGCCGGATGCTCCATCACAC -3'	60
		As	5'- GCCTTGCTCCCTAACAAAAGAGG -3'	

[a]AT, annealing temperature; [b]S, sense; [c]As, antisense
